# Case report: A third variant in the 5′ UTR of *TWIST1* creates a novel upstream translation initiation site in a child with Saethre-Chotzen syndrome

**DOI:** 10.3389/fgene.2022.1089417

**Published:** 2023-01-04

**Authors:** Francisca Diaz-Gonzalez, Javier M. Sacedo-Gutiérrez, Stephen R. F. Twigg, Eduardo Calpena, Fernando E. Carceller-Benito, Manuel Parrón-Pajares, Fernando Santos-Simarro, Karen E. Heath

**Affiliations:** ^1^ Institute of Medical & Molecular Genetics (INGEMM), Hospital Universitario La Paz, Universidad Autónoma de Madrid, IdiPAZ, Madrid, Spain; ^2^ Skeletal Dysplasia Multidisciplinary Unit (UMDE) and ERN-BOND, Hospital Universitario La Paz, Madrid, Spain; ^3^ Department of Neurosurgery, Hospital Universitario la Paz, Universidad Autónoma de Madrid, IdiPAZ, Madrid, Spain; ^4^ Clinical Genetics Group, MRC Weatherall Institute of Molecular Medicine, University of Oxford, John Radcliffe Hospital, Oxford, United Kingdom; ^5^ Department of Radiology, Hospital Universitario La Paz, Universidad Autónoma de Madrid, Madrid, Spain; ^6^ Centro de Investigación Biomédica en Red de Enfermedades Raras (CIBERER, U753), Instituto Carlos III, Madrid, Spain

**Keywords:** craniosynostosis, Saethre-Chotzen syndrome, Twist1, 5′ UTR, genetics

## Abstract

**Introduction:** Saethre-Chotzen syndrome, a craniosynostosis syndrome characterized by the premature closure of the coronal sutures, dysmorphic facial features and limb anomalies, is caused by haploinsufficiency of *TWIST1*. Although the majority of variants localize in the coding region of the gene, two variants in the 5′ UTR have been recently reported to generate novel upstream initiation codons.

**Methods:** Skeletal dysplasia Next-generation sequencing (NGS) panel was used for genetic analysis in a patient with bicoronal synostosis, facial dysmorphisms and limb anomalies. The variant pathogenicity was assessed by a luciferase reporter promoter assay.

**Results:** Here, we describe the identification of a third ATG-creating *de novo* variant, c.-18C>T, in the 5′ UTR of *TWIST1* in the patient with a clinical diagnosis of Saethre-Chotzen syndrome. It was predicted to create an out-of-frame new upstream translation initiation codon resulting in a 40 amino acid larger functionally inactive protein. We performed luciferase reporter promoter assays to demonstrate that the variant does indeed reduce translation from the main open reading frame.

**Conclusion:** This is the third variant identified in this region and confirms the introduction of upstream ATGs in the 5′ UTR of *TWIST1* as a pathogenic mechanism in Saethre-Chotzen syndrome. This case report shows the necessity for performing functional characterization of variants of unknown significance within national health services.

## Introduction

Craniosynostosis syndromes encompass a large group of heterogeneous conditions characterized by the premature closure of one or more cranial sutures, which leads to abnormal skull shape and distinctive craniofacial deformities. Their combined estimated incidence is approximately one in 2100 live births (Wilkie et al., 2017). Six genes, *FGFR2*, *FGFR3*, *TWIST1*, *EFBN1*, *TCF12* and *ERF,* account for approximately 84% of all genetically diagnosed cases (Paumard-Hernández et al., 2014; Wilkie et al., 2017; Hyder et al., 2021). Recently, a seventh gene has been added to this list, *SMAD6* (Calpena et al., 2020).

Heterozygous loss-of-function variants in *TWIST1* cause Saethre-Chotzen syndrome (SCS, MIM 101400), characterized by coronal suture synostosis, dysmorphic facial features, ptosis, hypertelorism and distal limb abnormalities such as brachydactyly or cutaneous syndactyly (El Ghouzzi et al., 1997; Howard et al., 1997).

*TWIST1*, located on chromosome 7p21.1 (Bourgeois et al., 1996), encodes a 202 amino acid class II basic helix-loop-helix (bHLH) transcription factor involved in calvarial development. TWIST1 plays an important role in the formation of the fronto-parietal boundary within the developing coronal suture (Merrill et al., 2006; Yen et al., 2010), and exerts an anti-osteogenic function preventing its premature closure (Yousfi et al., 2001; Bialek et al., 2004; Ting et al., 2009; Teng et al., 2018).

The major underlying pathogenic mechanism responsible for the craniofacial and limb anomalies observed in SCS patients is haploinsufficiency, usually caused by heterozygous variants affecting the coding region of the gene or structural variants, such as inversions or translocations encompassing *TWIST1* regulatory elements, leading to functional loss of one gene copy (Hyder et al., 2021; Hirsch et al., 2022).

However, two pathogenic variants, in the 5′ untranslated region (5′ UTR) of *TWIST1* have been recently reported in two patients with SCS (Zhou et al., 2018). Both were shown to create novel upstream initiation sites and a reduction in translation of *TWIST1*. Here, we report the clinical, genetics and functional characterization of a third variant in the 5′ UTR of *TWIST1*, in a patient with bicoronal craniosynostosis.

## Patient and methods

### Case report

**FIGURE 1:**
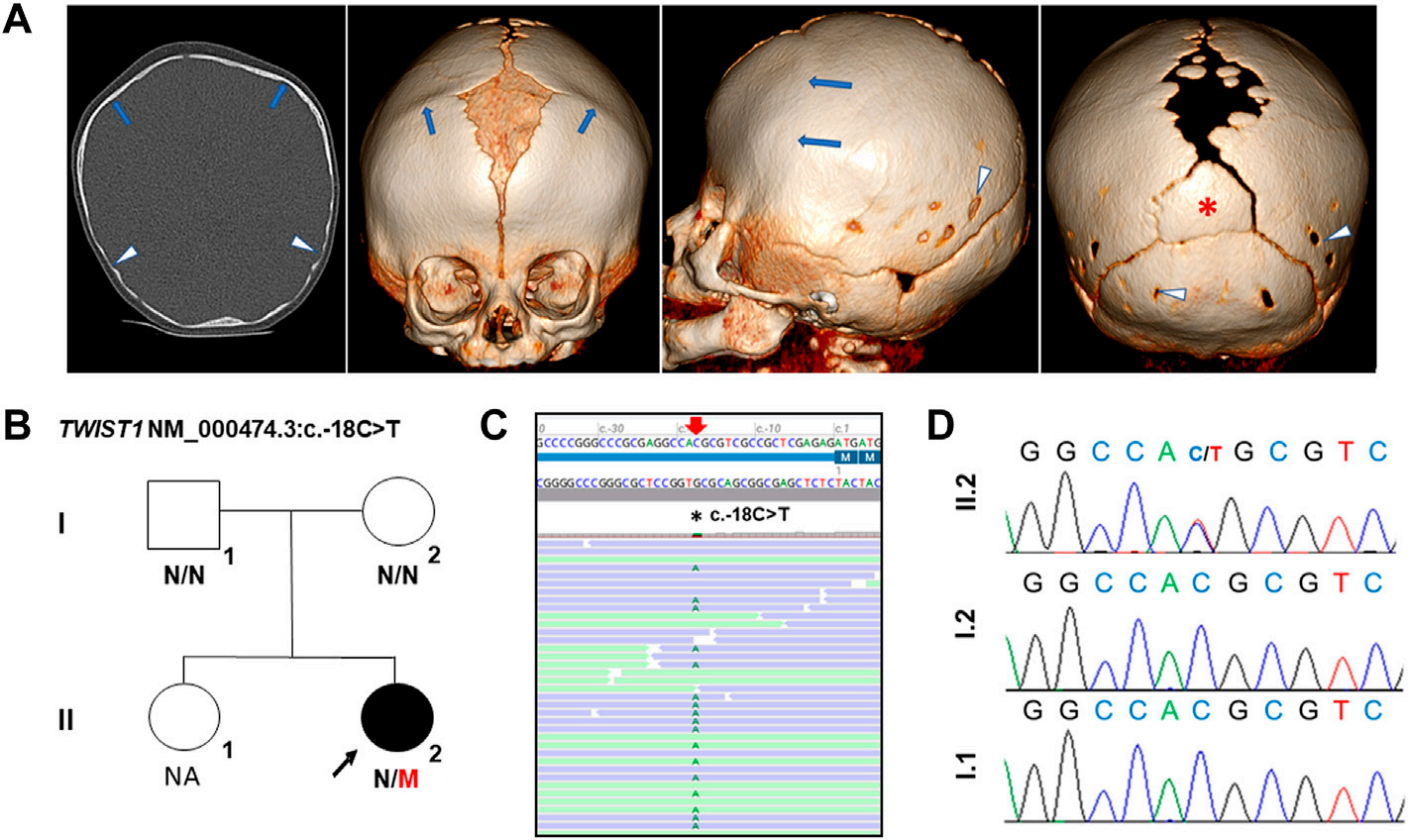
Clinical and genetic analysis of the proband with SCS. **(A)** Preoperative cranial CT images of the proband, age 3 months. From left to right: Frontal, lateral and axial images. Premature closure of both coronal sutures (blue arrows) is evident which lead to a brachycephalic cranial shape. Large anterior fontanel and superior part of metopic suture are present. Posterior part of sagittal suture and posterior fontanel are also enlarged, with several intrasutural bones. Note the presence of an inter-parietal or Inca bone (red asterisk). Posterior parietal bones and squamous part of occipital bone show thinning, parietal and occipital foramina (white arrow heads). **(B)** Pedigree of family. M, mutant; N, normal. **(C)** Alignment of NGS sequences of the proband highlighting the c.-18C>T variant. NGS panel: Average read depth 760X, 99.06% of bases >20X coverage and the variant reads 66:61 (reference:alternative). **(D)** Chromatograms showing the *de novo TWIST1* 5′ UTR variant, NM_000474.3:c.-18C>T in the proband. NA: Not available for genetic analysis.

The female proband, the second child of non-consanguineous healthy parents, was referred at 6 months of age from the neurosurgery department for molecular studies of craniosynostosis. She was born at term weighing 2500 g and craniosynostosis was noted. At 3 months old, cranial computed tomography (CT), performed prior to craniofacial surgery, revealed premature closure of both coronal sutures leading to an abnormal, brachycephalic head shape, large opening along the sagittal suture with a large anterior and posterior fontanel and several intra-sutural bones. An inter-parietal or Inca bone was also evident along with parietal and occipital foramina (Figure 1A). Physical examination showed frontal bossing, midface hypoplasia, mild pectus excavatum and slightly widened thumbs. No other skeletal anomalies were observed and neurological assessment was normal. No relevant familiar clinical history was reported. A clinical suspicion of Muenke syndrome (MIM 602849) or Saethre-Chotzen syndrome (MIM 101400) was suggested. The child underwent successful cranial surgery at 4 and 11 months old. The timeline of the case is described in supp table 1.

### Genetic analysis

The performed studies were reviewed and approved by the Hospital La Paz Ethical Committee (PI-5107). The participants provided their written informed consent to participate in this study.

Molecular screening of the proband was performed by a custom-designed NGS skeletal dysplasia gene panel (SKELETALSEQ.V11, n = 439 genes) using SeqCap EZ technology (Roche Nimblegen, Madison, WI, United States) and sequenced on a HiSeq platform (Illumina, San Diego, CA, United States). Analysis and variant filtering were performed as previously described (Sentchordi-Montané et al., 2021). The identified variant was subsequently validated by Sanger sequencing along with parental segregation.

### In silico prediction analysis of translation initiation site

Three online prediction tools: ATGpr (https://atgpr.dbcls.jp/), DNA functional site miner (DNAFSMiner, https://dnafsminer.bic.nus.edu.sg/) and NetStart (https://www.cbs.dtu.dk/services/NetStart/) were used to evaluate the potential initiation activity of the identified variant.

### Functional characterization of TWIST1 5′ UTR variants

#### Plasmid constructions

The psiCHECK-2 empty vector (EV), as well as the human *TWIST1* 5′ UTR wild type (psiCHECK2-TWIST1-5′UTR-WT) and *TWIST1* c.-263C>A positive control mutant (psiCHECK2-TWIST1-c.-263C>A) were as previously described (Zhou et al., 2018; Calvo et al., 2018). The c.-18C>T mutant was generated by site-directed mutagenesis of the WT vector following the manufacturer’s indications (QuikChange II Site-Directed Mutagenesis Kit, Agilent technologies). Mutagenesis primers (mutated base underlined) were 5′- GCC​CCG​GGC​CCG​CGA​GGC​CA **T** GCG​TCG​CCG​CTC​GAG​AGA​TG - 3' (forward) and the other being the reverse complement. Incorporation of the variant into the construct was verified by Sanger sequencing.

#### Cell culture, transient transfection and luciferase reporter assays

HEK293 cells were maintained in 1X Dulbecco’s modified Eagle’s medium (DMEM) supplemented with 10% fetal bovine serum and 1% penicillin/streptomycin (Gibco, Life technologies, CA, United States) at 37°C and 5% CO_2_. Twenty-4 hours prior to transfection, cells were seeded in 12-well plates at 2 x 10^5^ cells/well, and 2 µg of vector was transfected using FuGENE 6 (Promega, Madison, WI) at a 1:2 ratio of cDNA to FuGENE 6 following the manufacturer’s instructions.

HEK293 cells were transiently transfected either with the EV, the WT, the c.-18C>T or the c.-263C>A mutant as a positive control. Twenty-4 hours after transfection, cells were collected, lysed and the *Renilla* and *Firefly* luciferase activities were measured with the Dual-Luciferase^®^ Reporter assay (Promega, Madison, WI, United States) according to the manufacturer’s protocol. Each construct was transfected in triplicate, measured in triplicate and a total of three biological replicates were performed. Data are presented as percentages relative to WT and statistical analysis was performed by Student’s *t*-test with values considered significantly different at *p* < 0.05.

## Results

### Genetic and molecular findings

A heterozygous variant located in the 5′ UTR of *TWIST1* was identified in the proband: NM_000474.3:c.-18C>T p.? but absent from the parents (Figures 1B,C) (ClinVar SCV002589128). This variant is absent from large population databases (gnomAD, https://gnomad.broadinstitute.org) and has not been previously reported (HGMD Professional, Qiagen, CA, United States); ClinVar, www.ncbi.nlm.nih.gov/clinvar/LOVD, www.lovd.nl). The variant was classified as a variant of unknown significance (VUS) according to the recommendations of the American College of Medical Genetics and Genomics (Richards et al., 2015).

### In silico analysis of the c.-18C>T variant

**FIGURE 2:**
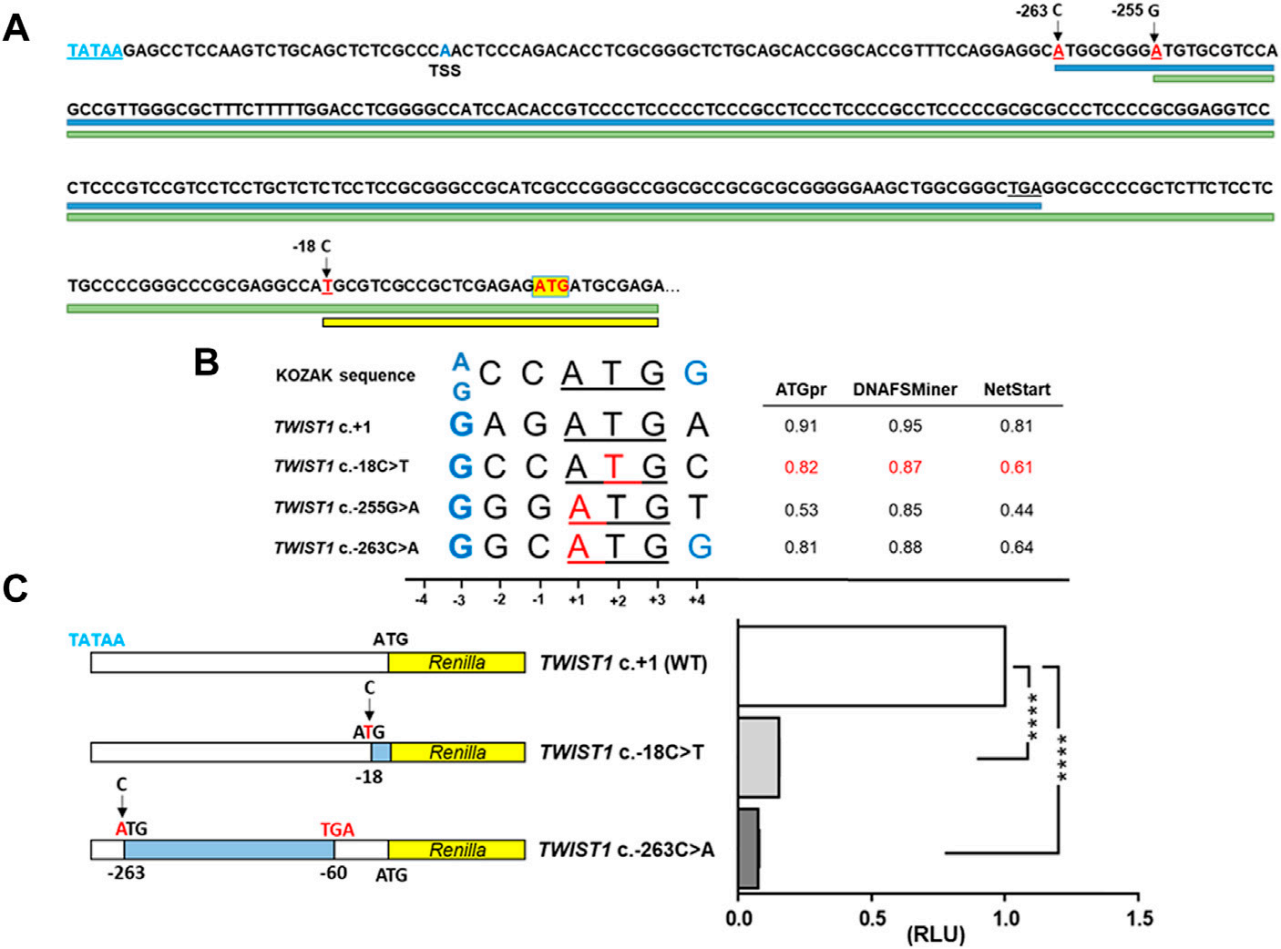
Effect on translation of the *TWIST1* 5′ UTR variant, c.-18C>T. **(A)** Genomic sequence showing the three upstream ATG-generating variants reported here and previously (mutated bases marked in red). Main wildtype *TWIST1* start codon (ATG) is indicated by a yellow box. Blue, green and yellow lines indicate the ORFs generated from the ATG sites of the c.-263, c.-255 and c.-18 variants respectively. TATAA box (blue letters) and transcription start site (TSS) are indicated **(B)** Alignment of the Kozak consensus sequence with the ATGs of the variant c.-18C>T, the *TWIST1* main start codon (+1) and the c.-255G>A and c.-263C>A variants. Mutated positions indicated in red. The important purine (G/A) at -3 from the ATG and a G at +4 are considered functionally critical (denoted in blue). In the right panel, evaluation of the translation initiation strength by ATGpr, DNAFSminer and NetStart, of the newly created upstream ATGs for the c.-18C>T variant (scores in red) compared to the WT sequence and the previously described positive controls. **(C)** Luciferase reporter DNA constructs psiCHECK2-*TWIST1*-5′UTR-WT, c.-18C>T and c.-263C>A positive control (left panel). The c.-263G>A variant creates an ORF of 68 codons that ends at a highly conserved stop codon at position c.-60 from the mORF (blue shading). The normalized luciferase reporter assay results of the WT, c.-18C>T mutant and the c.-263C>T positive control mutant are shown in the right panel. Each construct was transfected in triplicate, measured in triplicate in the luciferase assay and a total of three biological replicates were performed. RLU: relative luciferase units. The c.-18C>T mutant showed significantly reduced levels of *Renilla* activity (RLU: relative luciferase units) compared to WT (0.1537 ± 0.0010), similar to that observed with the c.-263C>A mutant (0.0759 ± 0.0041). RLU were significantly reduced (*p* < 0.0001) for both mutants compared to the WT.

The variant creates an ATG upstream the main open reading frame (mORF), so we evaluated whether it could create an alternative translation initiation codon by firstly determining if it alters the Kozak consensus sequence (Kozak, 1986). A purine (G/A) at -3 from the ATG is considered functionally critical and a G at +4 is preferred. The sequence flanking the c.-18C>T variant indeed has a G at -3 but not a G at +4 (Figure 2B). Three online tools: ATGpr, DNAFSMiner and NetStart, predicted the variant to create an alternative out-of-frame initiation site upstream of the *TWIST1* mORF (Figure 2B). Furthermore, if this aberrant transcript is translated then it would generate a mutant protein of 242 amino acids in size, entirely out of frame and using a stop codon 120bp downstream of the wildtype stop codon (ATPgr, https://atgpr.dbcls.jp/).

### Functional analysis of c.-18C>T variant on the translation of TWIST1 mORF

To test whether the *TWIST1* c.-18C>T variant affects protein translation from the mORF, we performed a dual-luciferase reporter assay in HEK293 cells transiently transfected with a construct containing the 5′ UTR of *TWIST1*. We compared luciferase activities for WT, c.-18C>T or the previously reported variant, c.-263C>A, as a positive control (Figure 2C). The c.-18C>T mutant showed significantly reduced levels of *Renilla* activity compared to WT (0.1537 ± 0.0010), similar to that observed with the c.-263C>A mutant (0.0759 ± 0.0041 Figure 2C).

## Discussion

SCS is one of the most common craniosynostosis syndromes. Although the vast majority of SCS causing variants are located within the coding region of *TWIST1* (Zhou et al., 2018), two pathogenic variants, c.-263C>A and c.-255G>A, have been identified in the non-coding regulatory region in the 5′ UTR of *TWIST1,* located in exon 1. Both variants create an alternative upstream ATG that affects translation of the *TWIST1* mORF (Zhou et al., 2018).

Here, we report a third pathogenic variant in the 5′ UTR of *TWIST1* (c.-18C>T), very close to the main start site, which is predicted to create an out-of-frame upstream ATG codon. The *de novo* variant was identified in a proband with SCS, who presented with bilateral coronal synostosis with large fontanels, intrasutural bones, and extensive parietal and occipital foramina (Figure 1A).

The context and the position of the ATG play a key role in the initiation of translation (Kozak, 1981). In eukaryotes, the flanking sequence of the ATG initiation codons has a conserved pattern of nucleotides known as the Kozak consensus (A/GCCATGG) (Kozak, 1986). Thus, the efficiency of an alternative upstream ATG to serve as translation initiation codon is influenced by the strength of the Kozak consensus. The optimal context requires a purine (A/G) at position -3 and a guanine at +4 is also preferable (Kozak, 1986) (Figure 2B). All three variants have a guanine at -3 of the Kozak sequence, whilst only one of the three, c.-263C>A has a guanine at +4. *In silico* analysis suggested that the c.-18C>T variant could act as potential competitor with the main ATG start site similarly to the c.-263C>A control mutant (Figure 2B).

The presence of an upstream ATG codon might reduce or abolish translation from the downstream main initiation site (Lomedico and McAndrew, 1982) so we tested whether the variant could reduce *TWIST1* translation by comparing the luciferase reporter activity in HEK293 cells transfected with either the c.-18C>T variant, WT or the previously reported variant c.-263C>A as a positive control (Zhou et al., 2018). We observed that *Renilla* luciferase activity was significantly reduced for the c-18C>T mutant compared to WT, similar to that observed for the positive control (>80% and >85%, respectively) (Figure 2C).

Previously, it was shown that the length of the upstream ORF and the distance between its stop codon and the *wild type* start initiation site are important for the repressive effect on translation of *TWIST1* (Zhou et al., 2018). Translation of the majority of eukaryotic mRNAs occurs by binding of the 43S preinitiation complex to the 5′ cap, and then scanning along the leader sequence in search for the first AUG codon available. Upstream ATGs and ORFs can also influence ribosome scanning efficiency, and thus modulate the translation levels of the main ORF (Iacono et al., 2005).

If translated, the c.-18C>T variant is predicted to result in an aberrant protein of 242 amino acids, while the c.-255G>A variant produces an 85 amino acid longer TWIST1 protein through the generation of an in-frame ATG. In contrast, the c.-263C>A variant creates an out-of-frame ORF which terminates in a stop codon located 59 bases upstream of the mORF. An *in vitro* transcription-translation assay and subsequent visualization of the protein products would determine if an aberrant protein is produced. If so, this aberrant mutant protein may be degraded or lack the dimerization and DNA binding capability.

With the three variants now identified to date in *TWIST1*, it is important that the entire 5′ UTR is screened for variants in patients with SCS, especially in genetically underdiagnosed SCS cases negative for coding or copy-number/structural variants. Although this region in *TWIST1* is exonic, it is a region that may be excluded from analysis for various reasons: it is not necessarily included in customized panel designs, bioinformatic analysis often excludes these regions and finally it is poorly covered in exomes. Pathogenic variants in the 5′ UTR of two other craniosynostosis genes also create upstream ORF-creating variants, *EFBN1* (Twigg et al., 2013; Romanelli Tavares et al., 2019) and more recently, *SMAD6* (Calpena et al., 2020). Thus, variants within the 5′ UTR of genes, and especially in haploinsufficient genes, should be carefully assessed in patients in whom the molecular defect has not been identified. Similar cases may be increasingly identified with the implementation of whole genome sequencing in healthcare but functional studies will be necessary to confirm the pathogenicity of these upstream ATG generating variants.

## Conclusion

We have identified a third upstream ORF-generating variant in the 5′ UTR of *TWIST1* in a child with SCS, which reduces the translation efficiency of the *TWIST1* mORF. The child now has a definitive clinical diagnosis which enables both appropriate clinical monitorization and genetic counselling. Thus, this case report shows the necessity for the creation of platforms/services for the functional characterization of variants of unknown significance within national health systems.

## Data Availability

The dataset for this article are not publicly available due to concerns regarding participant/patient anonymity. Requests to access the dataset should be directed to the corresponding author.
