# Supplementary material for: Case report: A third variant in the 5′ UTR of TWIST1 creates a novel upstream translation initiation site in a child with Saethre-Chotzen syndrome
Source: Front Genet. 2023 Jan 4;13:1089417. doi: 10.3389/fgene.2022.1089417 (PMC9845400; doi:10.3389/fgene.2022.1089417)
Supplement: Supplementary file 1 [file Table1.docx]

Supplementary Table 1: Timeline of the described case.

| **Age (months)** | **Event** |
| --- | --- |
| 0 | Female born at term weighing 2500 g, craniosynostosis was noted |
| 6 months | Cranial computed tomography (CT) revealed premature closure of both coronal sutures leading to an abnormal, brachycephalic head shape, large opening along the sagital suture with a large anterior and posterior fontanel and several intra-sutural bones. An inter-parietal or Inca bone was also evident along with parietal and occipital foramina |
| 4 months | First cranial surgery performed |
| 6 months | Referred from neurosurgery dept to clinical genetics dept. A clinical suspicion of Muenke syndrome or Saethre-Chotzen syndrome was suggested |
| 8 months | Molecular testing identified a *de novo* variant of unknown significance in the 5´UTR of TWIST |
| 11 months | Second cranial surgery performed |
| 24 months | Functional analysis confirmed that the identified variant was pathogenic and that the patient had Saethre-Chotzen syndrome. |
